# Supplementary material for: Performance of 18F-FDG PET/CT in Selecting Thyroid Nodules with Indeterminate Fine-Needle Aspiration Cytology for Surgery. A Systematic Review and a Meta-Analysis
Source: J Clin Med. 2019 Aug 28;8(9):1333. doi: 10.3390/jcm8091333 (PMC6780221; doi:10.3390/jcm8091333)
Supplement: Supplementary file 1 [file jcm-08-01333-s001.pdf]

**Supplemental data**

**Table S1:** Final diagnosis of included malignant thyroid nodules.

| First author, year | Papillary thyroid carcinoma (n) | Follicular thyroid carcinoma (n) | Hürthle cell carcinoma (n) | Medullary thyroid carcinoma (n) | Low differentiated carcinoma (n) | Anaplastic carcinoma (n) | Overall (n) |
|---|---|---|---|---|---|---|---|
| Mitchell, 2005 [34] | 1 | 0 | 0 | 0 | 0 | 0 | 1 |
| Hales, 2008 [35] | 8 | 0 | 0 | 0 | 0 | 0 | 8 |
| Deandreis, 2012 [36] | 7 | 3 | 0 | 0 | 0 | 0 | 10 |
| Muñoz Pérez, 2013 [37] | 9 | 0 | 3 | 1 | 0 | 0 | 13 |
| Merten, 2016 [38] | 4 | 1 | 2 | 0 | 0 | 0 | 7 |
| Pathak, 2016 [39] | 17 | 2 | 0 | 0 | 0 | 0 | 19 |
| Piccardo, 2016 [40] | 13 | 5 | 0 | 0 | 0 | 0 | 18 |
| Nguyen, 2018 [41] | 18 | 9 | 0 | 1 | 2 | 1 | 31 |
| Overall (n) | 77 | 20 | 5 | 2 | 2 | 1 | 107 |

Legend: In Nguyen, 2018 three malignant nodules had unknown $^{18}$F-FDG uptake and were excluded from the analysis.

**Table S2:** Final diagnosis of included benign thyroid nodules.

| First author, year | Follicular adenoma (n) | Nodular hyperplasia (n) | Hürthle cell adenoma (n) | Tumor with uncertain malignant potential (n) | Multi nodular goiter (n) | Lymphocy tic thyroiditis (n) | Other (n) | Overall (n) |
|---|---|---|---|---|---|---|---|---|
| Mitchell, 2005 [34] | 16 | 0 | 1 | 0 | 6 | 0 |  | 23 |
| Hales, 2008 [35] | 0 | 2 | 0 | 0 | 2 | 2 | 2 | 8 |
| Deandreis, 2012 [36] | 21 | 0 | 11 | 12 | 0 | 0 | 0 | 46 |
| Muñoz Pérez, 2013 [37] | 6 | 20 | 5 | 0 | 0 | 2 | 0 | 33 |
| Merten, 2016 [38] | - | - | - | - | - | - | - | 44 |
| Pathak, 2016 [39] | 23 | 0 | 8 | 0 | 0 | 0 | 0 | 31 |
| Piccardo, 2016 [40] | 21 | 48 | 0 | 0 | 0 | 0 | 0 | 69 |
| Nguyen, 2018 [41] | - | - | - | - | - | - | - | 77 |
| Overall (n) | 87 | 70 | 25 | 12 | 8 | 4 | 0 | 331 |

Legend: In Nguyen, 2018 four benign nodules had unknown $^{18}$F-FDG uptake and thus excluded from the analysis; - = not reported.

**Table S3:** Size of included thyroid nodules with indeterminate FNA.

| First author, year | Median (mm) | Range (mm) | Mean (mm) | Standard deviation (mm) |
|---|---|---|---|---|
| Mitchell, 2005 [34] | - | - | - | - |
| Hales, 2008 [35] | - | 1-53 | - | - |
| Deandreis, 2012 [36] | 21 | 10-57 | - | - |
| Muñoz Pérez, 2013 [37] | - | 10-85 | 28 | - |
| Merten, 2016 [38] | 18 | 8-74 | - | - |
| Pathak, 2016 [39] | - | - | 26 | 13 |
| Piccardo, 2016 [40] | 20 | 13-30 | - | - |
| Nguyen, 2018 [41] | 31 | 9-136 | - | - |

Legend: - = not reported.

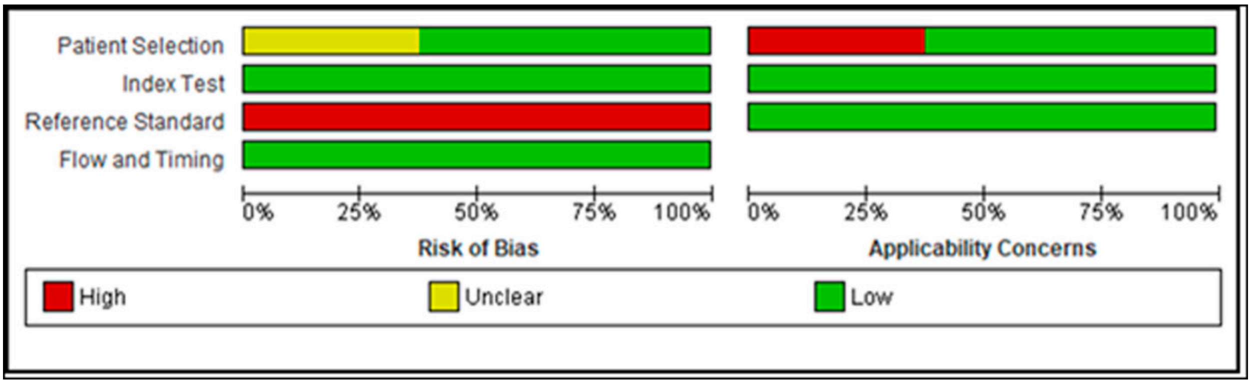


**Figure S1.** Risk of bias and applicability concerns graph: review authors' judgements about each domain presented as percentages across included studies.

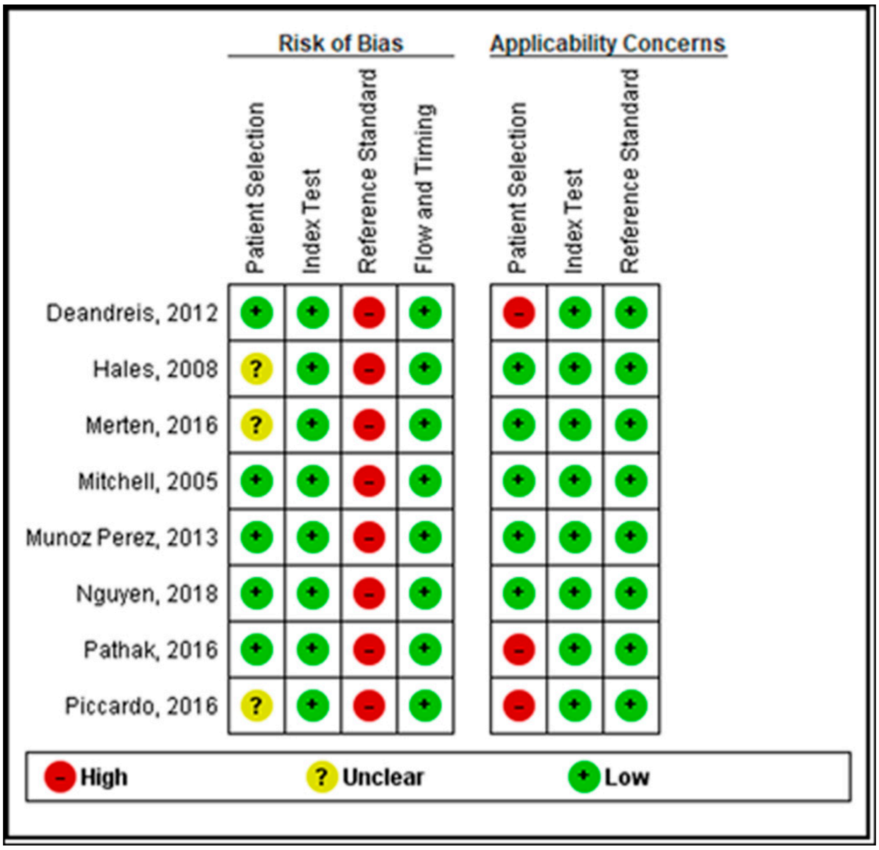


**Figure S2.** Risk of bias and applicability concerns summary: review authors' judgements about each domain for each included study.
